# *Coleus aromaticus* Benth.—A Plant with Strong Anticancer and Antioxidant Potential In Vitro

**DOI:** 10.3390/ph18111756

**Published:** 2025-11-18

**Authors:** Justyna Stefanowicz-Hajduk, Anna Hering, Rafał Hałasa, Szymon Masiak, Karolina Turczyn, J. Renata Ochocka, Monika Asztemborska

**Affiliations:** 1 Department of Biology and Pharmaceutical Botany, Medical University of Gdańsk, 80-416 Gdańsk, Poland; anna.hering@gumed.edu.pl (A.H.); renata@gumed.edu.pl (J.R.O.); 2 Department of Pharmaceutical Microbiology, Medical University of Gdańsk, 80-416 Gdańsk, Poland; rafal.halasa@gumed.edu.pl; 3 Faculty of Pharmacy, Medical University of Gdańsk, 80-416 Gdańsk, Poland; szymon.masiak@gumed.edu.pl (S.M.); karolinaturczyn@gumed.edu.pl (K.T.); 4 Institute of Physical Chemistry, Polish Academy of Sciences, 01-224 Warsaw, Poland; masztemborska@ichf.edu.pl

**Keywords:** gastric cancer cells, colorectal cancer cells, antimicrobials, gas chromatography, carvacrol, total phenolic content, total flavonoid content

## Abstract

**Background/Objectives**: Gastrointestinal cancers, including gastric and colon cancers, constitute a serious threat to global health due to their high incidence and limited treatment outcomes. Thus, natural products are becoming increasingly popular as potential chemopreventive agents. *Coleus aromaticus* Benth. is mainly used as a tasty addition to dishes and juices due to its aromatic and nutritional properties. The plant has many biological and pharmacological effects that require deeper evaluation. In this study, anticancer, antioxidant, and antimicrobial activities of ethanol, ethanol/water extracts, and juice from *C. aromaticus* leaves were evaluated. **Methods**: (3-[4,5-dimethylthiazol-2-yl]-2,5-diphenyltetrazolium bromide) MTT assay, DPPH (2,2-diphenyl-1-picrylhydrazyl), ABTS (2,2-azinobis-(3-ethylbenzothiazoline-6-sulfonate)), molybdenum reducing power assay, and broth microdilution technique were used, respectively. Additionally, total phenolic (TPC) and total flavonoid content (TFC) with basic phytochemical composition of volatile compounds by GC-MS and GC-FID were assessed. **Results**: The results indicate that the strongest anticancer activity was provided by the ethanol extract with IC_50_ values of 4.94 ± 0.48 and 24.99 ± 1.80 µg/mL on human gastric AGS cells and human colorectal HCT 116 cells, respectively. The antioxidant potential was also the highest for the ethanol extract with IC_50_ values of 13.34 ± 0.11 (ABTS), 22.90 ± 1.30 (DPPH), and 290.17 ± 4.23 µg/mL (molybdenum reducing power). Antimicrobial experiments revealed that ethanol and ethanol/water extracts were the most potent on *Clostridium perfringens* (MIC value was <0.02 mg/mL). Phytochemical analysis showed a significant content of phenolic and flavonoid compounds in the ethanol extract (75.87 ± 0.96 mg gallic acid equivalent/g dry extract and 176.01 ± 3.58 mg quercetin equivalent/g dry extract, respectively). Furthermore, all the extracts contained carvacrol (49.09, 28.15, and 25.68% of volatile fraction in ethanol, ethanol/water extracts and juice, respectively). Camphor and oleamide were also detected in large quantity. **Conclusions**: *C. aromaticus* can be considered as a potential agent in the prevention and treatment of gastrointestinal cancers, especially the ethanol extract from the plant leaves due to its strong anticancer and antioxidant properties.

## 1. Introduction

Gastric cancer remains one of the most serious health problems worldwide, ranking third among the leading causes of cancer-related deaths and the fifth most diagnosed malignancy worldwide. The disease is characterized by significant morphological heterogeneity, which makes it difficult to clearly classify and treat [1]. The main causes of the development of stomach cancer are a diet low in fruit and vegetables, age, and infection with *Helicobacter pylori*, which is a well-documented carcinogen in the case of this cancer [2]. *H. pylori* infection leads to chronic inflammation in the gastric mucosa, which plays a key role in initiating carcinogenesis [3].

In turn, colorectal cancer is the most common cancer occurring in the gastrointestinal tract, accounting for 13% of all emerging malignancies. The largest group of people suffering from this type of cancer are those aged 65–74, but this pathology is increasingly being diagnosed in younger patients due to factors related to a fast-paced modern lifestyle, such as poor nutrition, smoking, and a sedentary lifestyle. Currently, the progressive aging of the population is also negatively impacting the increasing incidence of colorectal cancer [4]. In the case of colon and rectal adenomas, therapy involves surgical resection of the tumor, the use of chemotherapy or radiotherapy, and often a combination of both. However, the insufficient effectiveness of the above therapies has forced scientists to look for alternative solutions. One such solution is research into the use of natural substances.

*Coleus aromaticus* Benth. is a plant from the Lamiaceae family native to Asia, Australia, and Africa, regions characterized by warm and even tropical climates. It may also be called *C. amboinicus* Lour., *Plectranthus amboinicus* (Lour.) Spreng., *P. aromaticus* Roxb., or *C. suganda* Blanco [5]. The common names for *C. aromaticus* depend on the region where the plant is used, for example, Poor man’s pork and Broad leaf thyme in Barbados, Jamaika thymian in Germany, and Puerto Rican oregano brujo and Cuban oregano in Puerto Rico [6]. Due to the content of compounds of high phytochemical importance, it has therapeutic properties used in folk medicine, e.g., in treating respiratory ailments such as asthma, bronchitis, the common cold, constipation, flu, headaches, and skin problems, as well as to increase breast milk production [6,7,8,9,10]. *C. aromaticus* can also be found in traditional regional cuisine as an ingredient in dishes or as a main spice. The plant leaves are well-known from their rich nutritional value due to the presence of vitamins, such as vitamin C (ascorbic acid) and B1 (thiamine). They are also a source of natural fiber, proteins, and minerals such as iron, zinc, calcium, potassium, and magnesium [11,12]. Therefore, the leaves are particularly used in many parts of the world. However, the focus should be on their proven biological and pharmacological effects, such as anti-inflammatory, antioxidant, antibacterial, antiviral, and even anticancer or antiepileptic properties [6,13,14,15,16,17,18]. For example, the ethanol extract of *C. aromaticus* leaves was tested on human lung cancer A549 cells. The extract reduced the cells viability, increased the production of reactive oxygen species (ROS) and the activity of caspase-3, and decreased NF-κB expression, along with modulating mRNA expression of pro-/anti-apoptotic genes (*Bax*, *Bad*, *Bcl-2*, and *Bcl-XL*) [19,20]. Next, the ethanol extract was used in a cytotoxicity assay on human hepatocellular carcinoma HepG2. A decrease in the cell viability and mitochondrial membrane potential, an increase in ROS production, disruption of nuclear morphology, and activation of caspase-8, -9, and -3 were observed [21,22]. Additionally, the hydroalcoholic extract from leaves of *C. aromaticus* showed anti-inflammatory and antitumor activities and inhibited the growth of sarcoma-180 and Ehrlich ascite carcinoma tumors in mice [15]. Gulla et al. synthesized and characterized silver nanoparticles using *C. aromaticus* leaf water extract and investigated their cytotoxicity, antibacterial, antimigratory, anti-inflammatory, and anticancer properties. They observed significant anticancer and antibacterial activity [23]. Another antibacterial effect of *C. aromaticus* was confirmed by Shubha et al. [24]. Those authors tested a hot water extract of *C. aromaticus* leaves and observed the inhibition of growth of *Escherichia coli* and *Salmonella typhimurium* and a stimulation of the growth of *Lactobacillus plantarum.* This can be applied for diarrhea and to accelerate the microbial gut balance during infection. Muniandy et al. tested an ethanol extract of *C. aromaticus* from leaves and roots against Gram-positive and Gram-negative microorganisms (*E. coli*, *Proteus mirabilis*, *Staphylococcus aureus*, *Pseudomonas aeruginosa*, *Klebsiella pneumoniae)* [25]. Another study used crude water, alcohol, Soxhlet water, and Soxhlet alcoholic extracts of *C. aromaticus* from leaves to screen for antibacterial activity against human respiratory pathogens isolated from sputum [26]. In turn, Murthy et al. examined the fungitoxic activity of *C. aromaticus* essential oil, which was found to be effective against various tested fungi—*Aspergillus flavus*, *A. niger*, *A. ochraceus* CFR 221, *A. oryzae*, *Candida versatilis*, *Fusarium* sp. GF-1019, *Penicillium* sp., and *Saccharomyces cerevisiae* [27].

Phytochemical studies of *C. aromaticus* composition revealed the presence of several dozen volatile (terpenes/terpenoids) and non-volatile compounds (polyphenols, vitamins, minerals, amino acids, tannins, alkaloids, saponins) [7]. However, the composition of the plant raw material is largely related to the plant’s climatic zone, harvest period, and identification methods, and therefore, some differences may occur. Additionally, volatile constituents of *C. aromaticus* obtained by distillation, hexane extraction, and supercritical carbon dioxide extraction methods had chemical differences [6,28]. Many of these volatile metabolites are important due to their anticancer and antibacterial properties [8,29,30]. Stimulation of the immune system and antibacterial properties also play an important role in the healing process of wounds, which heal faster with the use of this plant [31,32]. Several phytoconstituents like terpenoids, flavonoids, and vitamin C are well-known as promoters of the wound healing process [33,34,35]. In turn, the most important non-volatile components in the species include flavonoids and phenolic acids [6]. All these compounds are responsible for the antioxidant properties of the plant, which are mainly due to the structure of the phenolic metabolites [6,7,36]. The multiple antioxidant activity of water extract from *C. aromaticus* leaves has been proven by significant reducing power, superoxide scavenging ability, nitric oxide-scavenging activity, and also ferrous ion-chelating potency [17]. The hydroalcoholic extract of *C. aromaticus* resulted in a dose-dependent increase in radical scavenging ability against various free radicals [37]. The results of other studies showed that the alcoholic extract of the plant had remarkable antioxidant activity, directly linked to the concentration of the extract [38].

In this work, we estimated a wide range of biological activity of different *C. aromaticus* extracts—ethanol, ethanol/water, and juice prepared from fresh leaves. The extracts were tested on human gastric and colorectal cancer cell lines and selected microorganisms and evaluated for their antioxidant activity. Basic phytochemical analysis of the obtained extracts was performed as well.

## 2. Results

### 2.1. Cytotoxic Activity Assessment of C. aromaticus Extracts

**Figure 1:**
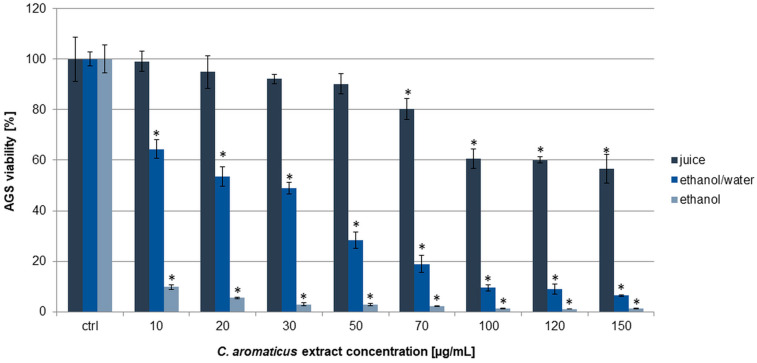
Viability of AGS cells after 24 h of incubation with *C. aromaticus* ethanol, ethanol/water extracts, and juice. The results were obtained via MTT assay and are presented as average values obtained from six repetitions in two independent experiments (*n* = 12). Error bars indicate standard deviations (±SD). Statistically significant results compared to the control (0.75% DMSO) are marked with an asterisk (“*”, *t*-Student’s test, *p* < 0.05).

To assess the cytotoxic effect of *C. aromaticus* extracts on AGS, HCT 116, and fibroblasts HFF-1, an MTT assay was used. The results obtained indicated that the ethanol extract had the strongest cytotoxic potential among the three tested extracts. The viability of gastric cancer cells was from 9.76 ± 1.06 to 1.23 ± 0.11% for the concentrations of 10 and 150 µg/mL, respectively [39]. The ethanol/water extract was less active, and the viability results were 64.39 ± 3.59, 53.51 ± 3.89, 48.89 ± 2.30, 28.33 ± 3.35, 18.86 ± 3.35, 9.48 ± 1.34, 9.00 ± 2.01, and 6.44 ± 0.27% for the extract concentrations of 10, 20, 30, 50, 70, 100, 120, and 150 µg/mL, respectively. The juice showed a cytotoxic effect above the concentration of 70 µg/mL. The viability of the cells at the juice concentrations of 70, 100, 120, and 150 µg/mL was 80.26 ± 4.14, 60.61 ± 3.87, 60.12 ± 1.37, and 56.53 ± 5.65%, respectively (Figure 1). The IC_50_ values for the ethanol and ethanol/water extracts were 4.94 ± 0.48 and 22.82 ± 1.82 µg/mL, respectively (Table 1).

**Figure 2:**
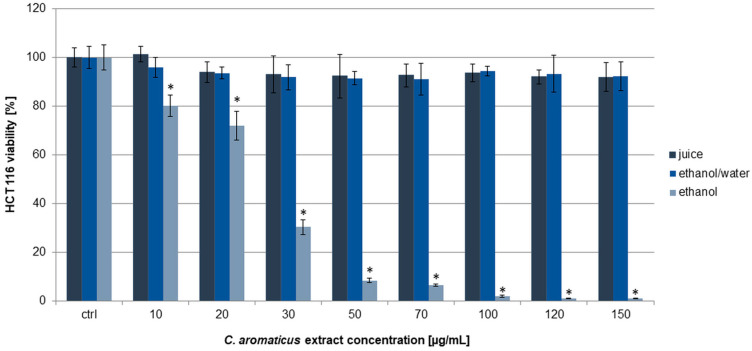
Viability of HCT 116 cells after 24 h of incubation with *C. aromaticus* ethanol, ethanol/water extracts, and juice. The results were obtained via MTT assay and are presented as average values obtained from six repetitions in two independent experiments (*n* = 12). Error bars indicate standard deviations (±SD). Statistically significant results compared to the control (0.75% DMSO) are marked with an asterisk (“*”, *t*-Student’s test, *p* < 0.05).

The HCT 116 cells turned out to be less sensitive to the action of all the extracts, especially in the case of *C. aromaticus* ethanol/water extract and juice (Figure 2). The strongest effect was for the ethanol extract with viability results of 80.13 ± 4.39, 71.99 ± 5.89, 30.36 ± 2.98, and below 10% for the concentrations of 10, 20, 30, and above 50 µg/mL, respectively. The IC_50_ value for the ethanol extract was 24.99 ± 1.80 µg/mL [39]. In the case of ethanol/water extract and juice, the viability results were at above 90% in all the used concentrations.

**Figure 3:**
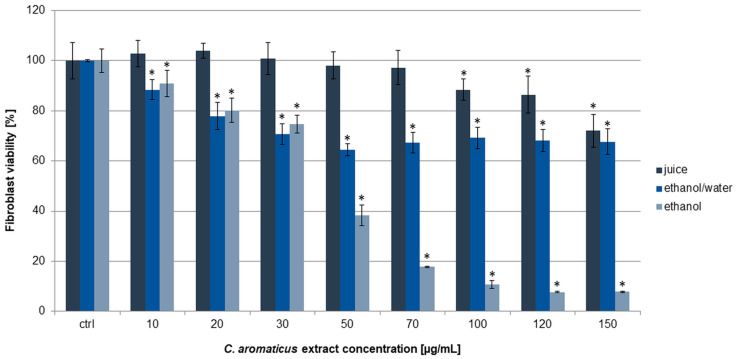
Viability of fibroblasts HFF-1 after 24 h of incubation with *C. aromaticus* ethanol, ethanol/water extracts, and juice. The results were obtained via MTT assay and are presented as average values obtained from six repetitions in two independent experiments (*n* = 12). Error bars indicate standard deviations (±SD). Statistically significant results compared to the control (0.75% DMSO) are marked with an asterisk (“*”, *t*-Student’s test, *p* < 0.05).

In this work, human fibroblasts HFF-1 were used as a noncancer control line. Similarly, ethanol extract was the most active on the cells, and the viability results were 90.98 ± 5.28, 80.18 ± 4.87, 74.68 ± 3.59, 38.43 ± 4.13, and below 20% for the concentrations of 10, 20, 30, 50, and above 70 µg/mL, respectively. The calculated IC_50_ value was 45.31 ± 2.28 µg/mL (Table 1). In the experiments with ethanol/water extract and juice, the viability was above 60 and 70%, respectively, in all the used concentrations (Figure 3). Interestingly, the ethanol extract at a concentration of 10 µg/mL did not show cytotoxic activity on fibroblasts, while on AGS cells, we observed a strong anticancer effect. A similar effect was obtained for the ethanol extract concentration of 30 µg/mL and colorectal cancer cells, where it showed significant cytotoxic activity against HCT 116 but not against fibroblasts.

### 2.2. Evaluation of Antioxidant Effect of C. aromaticus Extracts

Determination of the free radicals scavenging potential, as well as the reducing properties of the extracts obtained from *C. aromaticus*, was performed with the DPPH, ABTS, and molybdenum reducing power assays. In these tests, ascorbic acid was used as a standard. The ABTS test is considered a decolorization test, suitable for both lipophilic and hydrophilic antioxidants, including flavonoids, phenolic acids, and carotenoids. It measures the antioxidant capacity by a spectrophotometric method, where antioxidants neutralize the pre-formed stable blue/green ABTS radical cation (ABTS^•+^). When antioxidants react with this cation, they donate hydrogen and reduce it to a colorless form, leading to a decrease in absorbance at a specific wavelength. The degree of color loss is directly proportional to the antioxidant capacity of the sample. The principles of the DPPH assay are similar. In this test, a stable purple free radical (DPPH) reacts with an antioxidant, which reduces it to a stable non-radical yellow compound (DPPH-H). The color change from purple to yellow is directly proportional to the antioxidant’s ability to scavenge free radicals. In turn, the phosphomolybdenum reduction assay is a method for evaluating the antioxidant capacity of substances/extracts by measuring their ability to reduce Mo(VI) to Mo(V) and providing insights into their effectiveness in neutralizing free radicals.

**Figure 4:**
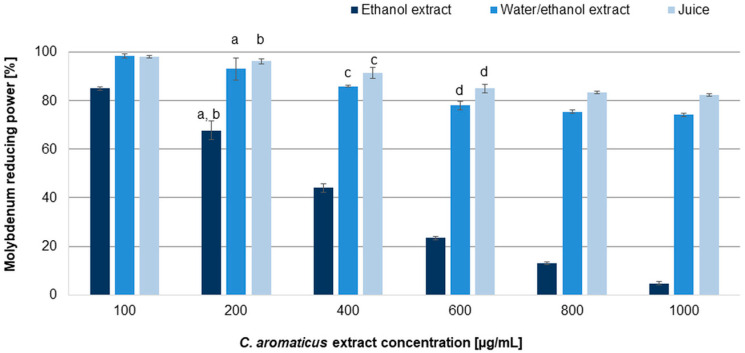
Molybdenum reducing power of *C. aromaticus* ethanol, ethanol/water extracts, and juice. Error bars indicate standard deviation (± SD) obtained from three independent experiments performed in three replicates (*n* = 9). Statistically significant differences among samples are indicated with “a”, “b”, “c”, and “d” within group (ANOVA with Tukey’s post hoc test, *p* < 0.05).

The results revealed moderate dose-dependent antiradical and reducing capabilities of the tested extracts. The IC_50_ values resulting from the tests are summarized in Table 2. Among the analyzed extracts, the best, and similar to ascorbic acid, ability to scavenge free radicals was indicated for the ethanol extract (IC_50_ values: 22.90 ± 1.30 and 13.34 ± 0.11 µg/mL for the DPPH and ABTS assays, respectively) [39], while significantly weaker properties were demonstrated for the ethanol/water extract (IC_50_ values: 418.40 ± 2.75 and 79.94 ± 0.55 µg/mL for the DPPH and ABTS assays, respectively). Juice indicated very low capability to scavenge free radicals (IC_50_ values: 879.68 ± 2.55 and 148.90 ± 1.45 µg/mL for the DPPH and ABTS assays, respectively). The reduction test (Figure 4) confirmed the potential of the ethanol extract (IC_50_ value 290.17 ± 4.23 µg/mL). Although all tested extracts increased the reduction in molybdenum with increasing concentration, the juice and the ethanol/water extract did not achieve the IC_50_ value (the study was carried out to a final concentration of 1000 µg/mL; higher concentrations caused the formation of a precipitate that prevented spectrophotometric measurement). The results observed for the ethanol extract showed that it was able to reduce molybdenum significantly, and despite being weaker than ascorbic acid, it acted almost completely at a concentration of 1000 µg/mL.

Antioxidant capabilities are closely related to the metal chelating activities of natural compounds and extracts. Thus, in our preliminary experiments, we estimated the chelating properties of all the tested extracts of *C. aromaticus* and their ability to bind Cu^2+^ ions. The results indicated that the extracts formed complexes with copper ions, and as a result, they can prevent the production of harmful free radicals. The complexation abilities of the extracts towards Cu^2+^ were evaluated based on shifts between the maximum absorption of solutions before an addition of CuSO_4_ and the maximum absorption of the respective solutions after addition of this salt [40]. Generally, if a shift is observed, it means that the ion chelation process has occurred. In this work, we obtained visible shifts of the maximum absorption in all cases (Figure S1, Supplementary Materials).

### 2.3. Antimicrobial Effect of C. aromaticus Extracts

To estimate the antibacterial and antifungal effects, we used different bacteria strains and *Candida albicans*. The obtained results showed generally weak activity of *C. aromaticus* ethanol, ethanol/water extracts, and juice on the tested microorganisms, except *Clostridium perfringens*. The strongest activity was observed for the ethanol extract and *Streptococcus*, *Staphylococcus* species, *Shigella sonnei*, *Yersinia enterocolitica*, *Helicobacter pylori*, and *Clostridium* species [39]. Among *Clostridium* strains, *C. perfringens* was the most sensitive to the ethanol and ethanol/water extracts. The MIC (minimum inhibitory concentration) and MBC (minimum bactericidal concentration) values were below 20 µg/mL (Table 3).

### 2.4. Phytochemical Analysis of C. aromaticus Extracts

#### 2.4.1. Content of Phenolic Compounds

The total phenolic content (TPC) was determined using the Folin–Ciocalteu spectrophotometric method. The reagent, which exhibits a yellow coloration, reacts with phenolic compounds by transferring electrons to the complex, which results in a blue coloration. The TPC for the *C. aromaticus* extracts is presented in Table 4. The TPC values were 27.19 ± 0.15, 35.13 ± 1.18, and 75.87 ± 0.96 mg gallic acid equivalent (GAE)/g dry extract (DE) for the juice, ethanol/water, and ethanol extracts, respectively. These results may be explained by the higher number of phenolic compounds extracted by pure ethanol. The flavonoid content of the extracts was expressed as milligrams of quercetin equivalent per gram of dry extract (mg QE/g DE), and the results are presented in Table 4. The tested probes indicated a variable content of flavonoids, probably related to the content of polar -OH groups in the ethanol/water extract (26.59 ± 0.52 mg QE/g DE) and ethanol extract (176.01 ± 3.58 mg QE/g DE). A negligible content of flavonoid compounds was found in the juice (12.90 ± 1.54 mg QE/g DE). The ethanol extract contained far higher amounts of phenolic and flavonoid compounds than the juice and ethanol/water extract.

Preliminary qualitative analysis with HPLC revealed the presence of rosmarinic acid, caffeic acid, gallic acid, and luteolin in the ethanol and ethanol/water extracts and gallic acid in the juice (Figure S2, Supplementary Materials).

#### 2.4.2. Compounds Identified in *C. aromaticus* Extracts by GC Analysis

**Figure 5:**
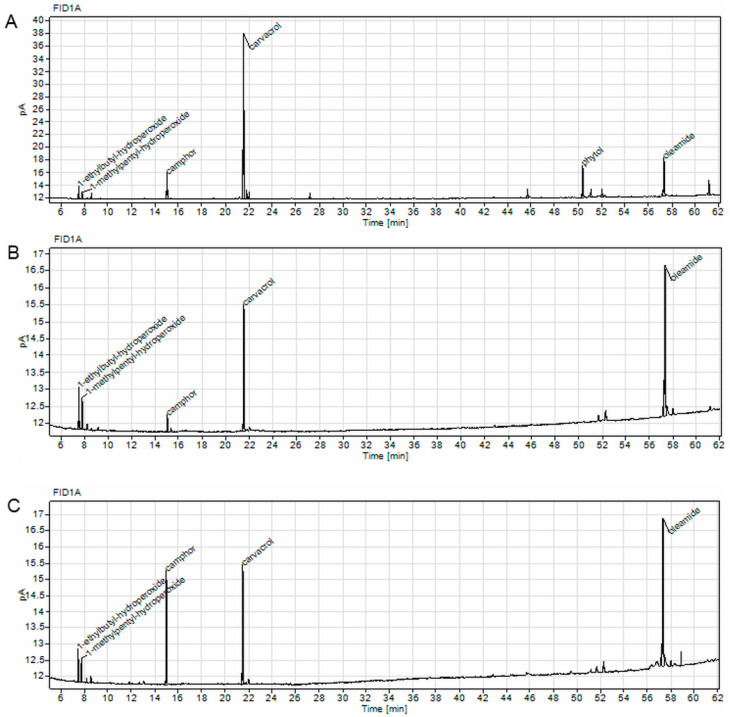
Chromatograms of *C. aromaticus* ethanol (**A**) and ethanol/water (**B**) extracts and juice (**C**) obtained with Agilent 8860 GC system equipped with FID detector.

To analyze the volatile compounds in the *C. aromaticus* ethanol, ethanol/water extracts, and juice, we performed GC experiments and identified the main secondary metabolites in the samples. The most abundant compound in the ethanol extract was carvacrol (49.09%), whereas in the ethanol/water extract and juice, oleamide was dominant (45.72 and 43.08%, respectively). In the juice, we also observed a large quantity of camphor (23.08%) [39], the largest amount among the tested extracts (Table 5 and Figure 5).

## 3. Discussion

In this study, we estimated a wide range of biological activity of *C. aromaticus* extracts. The experiments using the MTT assay revealed that the ethanol extract had the strongest potential on human gastric cancer AGS and colon cancer HCT 116 cells. In addition, we observed strong activity of the ethanol/water extract, but only towards AGS cells. What is important, the ethanol extract did not show cytotoxic activity on fibroblasts at a concentration of 10 µg/mL, while on AGS cells it exerted a strong anticancer effect. A similar effect was observed for the ethanol extract concentration of 30 µg/mL and HCT 116 cells, where it showed significant cytotoxic activity but not against fibroblasts. The ethanol/water extract also had a strong effect on AGS cells without high toxicity towards fibroblasts. In turn, the antimicrobial activity of *C. aromaticus* extracts was overall moderate. The strongest effect was observed for the ethanol extract and *Streptococcus*, *Staphylococcus* species, *Shigella sonnei*, *Yersinia enterocolitica*, *Helicobacter pylori*, and *Clostridium* species. Among *Clostridium* strains, *C. perfringens* was the most sensitive to the ethanol and ethanol/water extracts. It has been proven that the increase in some of the bacterial strains studied here (*E. coli*, *H. pylori* or *E. faecalis*) is associated with the release of pro-inflammatory and carcinogenic mediators and, consequently, with the presence of chronic inflammation and the risk of the development of gastrointestinal cancers [2,41].

These activities, both anticancer and antimicrobial, are strictly connected with the phytochemical composition of the extracts. The qualitative and quantitative analysis revealed that the most biologically active ethanol extract had a large amount of carvacrol—almost 50% in its volatile fraction. It is well-known that carvacrol has strong anticancer properties by modulating multiple molecular pathways governing apoptosis, metastasis, angiogenesis, and inflammation, including gastrointestinal cancers [42].

Carvacrol, as one of the main components of *C. aromaticus* essential oil and extracts, was examined on human melanoma cancer A375 cells, and it inhibited their growth and induced apoptosis. The cleavage of PARP and reduction in *Bcl-2* gene expression was observed in that work [30]. Another study showed that carvacrol inhibited cell proliferation and induced apoptosis in AGS cells by ROS generation, DNA damage, upregulation of Bax, caspase-3, and caspase-9 proteins, and downregulation of Bcl-2 in a dose-dependent manner [43].

Among all the tested extracts, ethanol extract also had the highest content of phenolic and flavonoid compounds. Preliminary HPLC analysis showed the presence of rosmarinic acid, caffeic acid, gallic acid, and luteolin. All these compounds may influence the anticancer effects of *C. aromaticus* extracts with varying strength. For example, luteolin prevents cancer via modulation of numerous pathways—inactivates proteins, such as procaspase-9, CDC2, and cyclin B or upregulates caspase-9 and caspase-3, cytochrome C, cyclin A, CDK2, and APAF-1. It also enhances the phosphorylation of p53 and the expression level of the *p53*-targeted downstream gene. The metabolite decreases VEGF and Bcl-2 expression and induces cell cycle arrest and apoptosis [44]. Furthermore, luteolin reduces the nuclear factor E2-related factor 2 (Nrf2) expression at both the mRNA and protein levels in NSCLC A549 cells [45]. Gallic acid stimulates apoptosis in tumor cells and suppresses their migration with influence on PI3K/Akt, ERK, and NF-κB pathways. The compound also enhances the sensitivity of tumor cells to chemotherapy [46]. Caffeic acid has demonstrated the ability to trigger apoptosis in cancer cells by influencing the mitochondrial pathway. Furthermore, it can inhibit NF-κB, STAT3, and ERK1/2, thereby reducing inflammatory responses, and activates the Nrf2/ARE pathway to enhance antioxidant cell defenses [47]. Natural compounds may inhibit Nrf2 signaling in cancer cells with high Nrf2 activity or can be Nrf2 inducers to protect normal cells from carcinogens [48].

The antimicrobial activity of the extract is most likely due to the absorption of polyphenols to bacterial membranes and their subsequent disruption and leakage of cellular contents. Luteolin can inhibit FAS II and DNA topoisomerase and interact with liposomal membrane in Gram-positive and Gram-negative bacteria [49,50]. Gallic acid disrupts the cytoplasmic membrane, induces intracellular potassium release and intracellular leakage, and inhibits the motility of bacteria [49,51]. Additionally, rosmarinic acid has a bacteriostasis function, which can destroy bacterial cells and cell proteins and inhibit the activity of Na^+^/K^+^-ATP-ase [52]. Volatile compounds of the plant essential oil—mostly monoterpenes and sesquiterpenes (camphor, carvacrol, thymol, caryophyllene)—are responsible for the antibacterial and antifungal activities. Due to the highly lipophilic nature and low molecular weight of terpenes/terpenoids, they are able to disrupt the cell membrane or inhibit the sporulation and germination of food spoilage fungi [29,53].

In our study, three antioxidant tests were used to assess the antioxidant potential of the extracts from *C. aromaticus*. The ABTS and DPPH tests provide information on the radical-scavenging abilities of the tested extracts, while the molybdenum test assesses their reducing abilities, which are related to the total antioxidant power. ABTS and DPPH radicals are commonly used tests, also utilized in other works to assess the antioxidant properties of *C. aromaticus* extracts. These tests revealed significant differences in the free radical scavenging abilities of the plant’s extracts due to variability in the composition of the main components, which depends on the type of material, solvent, and extraction method [54,55]. The influence of the solvent facilitates the dissolution of active ingredients contained in plant cells. The choice of ethanol in extraction procedures is conditioned by its low toxicity and ease of removal from the extract without leaving toxic residues. In this study, the obtained results indicated significantly lower antioxidant values for the juice, with slightly better values for the ethanol/water extract. By far, the best results were obtained for the ethanol extract. The demonstrated total reduction capacities (by molybdenum reducing power assay) of the *C. aromaticus* extracts were relatively low, even in the case of the ethanol extract, although this one reached the IC_50_ value. Molybdenum blue, as a spectrophotometric method, is widely used to estimate the antioxidant activity of natural products. However, this method can promote the degradation of plant metabolites, especially phenols and flavonoids, and the degradation products can interfere with the total antioxidant activity of extracts resulting in unchanging, increasing, or decreasing values [56]. Nevertheless, in our work, the results obtained in the molybdenum reducing power test are consistent with those in other assays and showed that among the tested samples, the ethanol extract had the highest antioxidant potency. This capacity was also confirmed by the chelating abilities of *C. aromaticus* extracts. Generally, plant extracts and natural compounds such as polyphenols can have chelating properties by binding metal ions—lead, copper, iron, cadmium, aluminum, and zinc—to form complexes. They can remove pro-oxidant toxic metals from the body, which cause cell membrane, lysosomes, nucleus, and mitochondria damage. Thus, they play a significant role in detoxification processes [57,58,59].

Compounds with strong free radical scavenging abilities can belong to various chemical groups. They generally require an unsaturated bond or a significant number of –OH groups [60]. The highest total phenolic and flavonoid content, indicated for the ethanol extract, confirmed that conclusion. Next, phytol-unsaturated diterpene, detected in this kind of extract, is known for antioxidant properties [61,62]. In addition, the antioxidant ability is also confirmed for carvacrol, though the presence of the –OH, related to the aromatic ring [63,64] and camphor [65]. These compounds might be responsible for the antiradical ability of the extracts. Phenolic compounds and flavonoids are well-known antioxidants that can prevent many diseases [66] by stimulation of the immune system, reduction in inflammation processes, and their antibacterial and even anticancer properties [67,68]. *C. aromaticus* extracts were characterized in the literature by high variability in the content of polyphenols, similar to the antioxidant activity, and the TPC and TFC depended on the solvent used or the extraction method [55,69,70]. According to our results, the ethanol extract from *C. aromaticus* presents high, similar to ascorbic acid, antioxidant properties with relatively good amounts of phenolics and flavonoids. The plant can play a significant role in protection of cancer at higher doses, while at lower doses, it may have high potential for treatment. This dose-dependent complex relationship is the result of the interaction of the plant compounds and may vary depending on the type of cells. Therefore, further research should be carried out to confirm the qualitative and quantitative composition of *C. aromaticus* extracts as well as the biological activity, especially that related to antioxidant, anti-inflammatory, and anti-mutagenic effects [71,72].

## 4. Materials and Methods

### 4.1. Plant Material and Extraction

*C. aromaticus* was obtained from a commercial garden (Garden Center Justyna, Gdańsk, Poland) and was deposited in the GDMA Herbarium (Herbarium of the Medical University of Gdańsk, Gdańsk, Poland, No. 4639). Fresh plant leaves (100 g each) were macerated with 95% or 50% ethanol (0.5 L; Merck Millipore, Burlington, MA, USA) for 24 h at room temperature. The juice was mechanically squeezed from the third part of the leaves. All the extracts were filtered, concentrated under reduced pressure at 40 °C, and lyophilized. The dry extracts were dissolved in DMSO (for antimicrobial and cytotoxic analyses; Merck Millipore, Burlington, MA, USA) or methanol (for antioxidant tests and TPC/TFC analyses; Merck Millipore, Burlington, MA, USA) and stored in the dark at 4 °C until further analysis.

### 4.2. Evaluation of Cytotoxic Activity of C. aromaticus Extracts

#### 4.2.1. Cell Lines

The human gastric AGS and colorectal HCT 116 cancer cell lines were obtained from the American Type Culture Collection (ATCC, Manassas, VA, USA). Human fibroblasts HFF-1 were obtained from LGC Standards (Teddington, Middlesex, UK). The cells were cultured in Dulbecco’s Modified Eagle’s Medium/Nutrient Mixture F-12 Ham, McCoy’s Medium with L-glutamine, and Fibroblast Growth Medium with Supplement Mix, respectively (Merck Millipore, Burlington, MA, USA). Penicillin (100 units/mL), streptomycin (100 µg/mL), and fetal bovine serum (FBS, 10% (*v*/*v*)) (Merck Millipore, Burlington, MA, USA) were added to the media. The cells were incubated at 37 °C and 5% CO_2_.

#### 4.2.2. MTT Assay

To assess the cytotoxic effect of three different extracts, the MTT assay was used. Briefly, the cells were seeded in 96-well plates (5 × 10^3^/well) and incubated for 24 h. The extracts were added to the cells in a concentration range of 10–150 µg/mL. The concentration of DMSO (solvent of the extracts) was 0.75% (*v*/*v*). After 24 h, the MTT reagent (Merck Millipore, Burlington, MA, USA) was added to the plates. The formazan crystals were dissolved in DMSO, and the absorbance was measured at 570 and 650 nm (Epoch, BioTek Instruments, Santa Clara, CA, USA). Six repetitions in at least two experiments were performed. The data are expressed as the percentage of cell viability and IC_50_ values (µg/mL) calculated in GraFit 7.0 software (Erithacus Software, West Sussex, UK).

### 4.3. Antioxidant Tests and Estimation of Metal Ion Complexation

#### 4.3.1. Materials

ABTS (2,2-azinobis-(3-ethylbenzothiazoline-6-sulfonate)), DPPH (2,2-diphenyl-1-picrylhydrazyl), ascorbic acid, gallic acid, quercetin, AlCl_3_, Na_2_CO_3_, Folin–Ciocalteu solution, monopotassium phosphate, and ammonium heptamolybdate were sourced from Merck Millipore (Burlington, MA, USA). EDTA, anhydrous copper (II) sulfate (VI) (CuSO_4_), hydrochloric acid (HCl), and sodium hydroxide (NaOH) were sourced from Avantor Performance Materials Poland S.A., Gliwice, Poland.

#### 4.3.2. Methods

Spectrophotometric assays were used to determine the radical scavenging ability and reducing properties of the tested extracts [73,74]. The introduced modifications were utilized in analysis with a 96-well plate and a spectrophotometer (Epoch, BioTek System, Winooski, VT, USA) [75]. In the experiments, ascorbic acid was used as a standard. For calculation of the IC_50_ values of the extracts, GraphPad Prism 9 software (version 9.0.0, GraphPad Software, San Diego, CA, USA) was used. For the analyses, a stock solution of a single extract (10 mg in 1 mL of 50% methanol (*v*/*v*)) was prepared and stored at 4 °C. Dilutions were conducted immediately before each analysis. The assays were performed in triplicate; three replications were performed in each analysis (*n* = 9). Solutions of radical mixtures with 50% methanol (*v*/*v*) were used as a negative control.

##### ABTS Assay

The ABTS radical scavenging activity of the *C. aromaticus* extracts was determined by the method described by Kwon et al., with modifications introduced by the authors [73,75]. In brief, 25 µL of different concentrations of the extracts were mixed with 25 µL of water and 150 µL of ABTS solution. After 30 min of incubation at 30 °C, the absorbance was analyzed at λ = 750 nm.

##### DPPH Assay

The DPPH radical scavenging activity of the *C. aromaticus* extracts was determined by the method described by Kwon et al., with modifications introduced by the authors [73,75]. In brief, 100 µL of different concentrations of the extracts were mixed with 100 µL of 0.06 mM DPPH (methanolic solution). The reaction mixtures were kept in the dark at room temperature. After 30 min. of incubation, the absorbance was analyzed at λ = 517 nm.

##### Molybdenum Reduction Assay

The reducing power of the *C. aromaticus* extracts was assessed using the phosphomolybdenum method [74] with slight modifications [75]. The extracts and reaction mixture were incubated at 90 °C. After 90 min, the samples were cooled to room temperature. In the next step, the absorbance was measured at 700 nm against a blank sample. The reducing power of the extracts was estimated from the ascorbic acid calibration curve.

#### 4.3.3. Cu^2+^ Ion Complexation by *C. aromaticus* Extracts

In order to estimate chelating ability of *C. aromaticus* juice and extracts, Cu^2+^ ions were used. The solution CuSO_4_ at a concentration of 0.5 mM was prepared (methanol:water, 7:3, *v*/*v*). The extracts and EDTA solution (a control) were prepared at a concentration of 0.1% (in methanol:water, 7:3) and divided into two groups. In the first group, the pH of the tested solutions was adjusted to 2 with HCl. In the second group, NaOH was used to obtain pH 5.5 of the solutions. The experiment was performed in a 96-well plate; UV–Vis absorption spectra of the extracts alone and after addition of Cu^2+^ were measured in the range 200–900 nm (Epoch, BioTek System, Winooski, VT, USA). The experiment was performed in triplicate.

### 4.4. Antimicrobial Activity

#### 4.4.1. Microorganisms

*Streptococcus* β-hemolytic group A PCM465, (PCM—Polish Collection of Microorganisms), *Enterococcus faecalis* ATCC1299, *Staphylococcus aureus* ATCC6538, *Staphylococcus aureus* ATCC43300 MRSA, *Escherichia coli* ATCC8739, *Salmonella enterica* ATCC13076, *Shigella sonnei* ATCC25931, *Yersinia enterocolitica* ZMF4195, *Corynebacterium diphtheriae* ZMF (collection of the Department of Pharmaceutical Microbiology, Medical University of Gdańsk, Gdańsk, Poland), *Listeria monocytogenes* PCM2191, *Clostridium sporogenes* ATCC19404, *Clostridium bifermentans* ATCC638, *Clostridium perfringens* ATCC13124, *Helicobacter pylori* ATCC43504, *Campylobacter jejuni* ZMF (collection of the Department of Pharmaceutical Microbiology, Medical University of Gdańsk, Gdańsk, Poland), *Lactobacillus acidophilus* PCM2499, *Lactobacillus paracasei* PCM2639, and *Candida albicans* ATCC26790 were used.

Brain–heart infusion broth (BHI, Becton Dickinson, Franklin Lakes, NJ, USA) supplemented with 10% bovine serum was used for the *Streptococcus* β-hemolytic group A and *Listeria monocytogenes* (strains growth in GENbag CO_2_, BioMerieux, Marcy-l’Étoile, France at 37 °C for 48 h). Brain–heart infusion broth (BHI, Becton Dickinson, Franklin Lakes, NJ, USA) supplemented with 10% bovine serum was used for *Clostridium sporogenes*, *Clostridium perfringens*, and *Clostridium bifermentans* (strains growth in GENbag anaerobe, BioMerieux, Marcy-l’Étoile, France at 37 °C for 48 h). *Staphylococcus aureus*, *Staphylococcus aureus* MRSA, *Escherichia coli*, *Salmonella enterica*, *Shigella sonnei*, and *Yersinia enterocolitica* were grown in Mueller–Hinton broth (cation-adjusted MH, Becton Dickinson, Franklin Lakes, NJ, USA) in an aerobic atmosphere at 37 °C for 48 h. *Corynebacterium diphtheriae* was incubated in brain–heart infusion broth (BHI, Becton Dickinson, Franklin Lakes, NJ, USA) supplemented with 10% bovine serum in an aerobic atmosphere at 37 °C for 72 h. *Helicobacter pylori* and *Campylobacter jejuni* were grown in BHI supplemented with 10% bovine serum in a microaerophilic atmosphere at 37 °C for 72 h (GENbag microaer, BioMerieux, Marcy-l’Étoile, France. *Candida albicans* was grown in Sabourauda broth (Sb, Becton Dickinson, Franklin Lakes, NJ, USA) in an aerobic atmosphere at 37 °C for 72 h. *Lactobacillus acidophilus* and *Lactobacillus paracasei* were incubated in MRS Broth in GENbag CO_2_, BioMerieux (Marcy-l’Étoile, France), at 37 °C for 48 h. After determination of the bacterial viability, BHI blood agar plates, MH agar plates, Sb agar, and MRS agar plates were used [76].

#### 4.4.2. Antibacterial Assay

The microorganism culture for the experiments was prepared by transferring cells from the stock cultures to a tube with the appropriate broth as described above. They were incubated with agitation for 24 h at 37 °C. The cultures were diluted with adequate broth to achieve an optical density corresponding to 10^6^ colony forming units per mL (CFU/mL) for bacteria species and 10^3^ CFU/mL for *Candida albicans* (except *Corynebacterium diphtheriae*, *Campylobacter jejuni*, *Helicobacter pylori*, *Clostridium sporogenes*, *Clostridium perfringens*, and *Clostridium bifermentans*). For *Corynebacterium diphtheriae*, *Campylobacter jejuni*, *Helicobacter pylori*, *Clostridium sporogenes*, *Clostridium perfringens*, and *Clostridium bifermentans,* the inoculum was prepared from bacterial colonies grown on BHI blood agar plates that had been incubated for 48 h in appropriate conditions with a final inoculum concentration of approximately 10^6^ CFU/mL [77,78]. The minimum inhibitory concentration (MIC) was determined by broth microdilution technique using 96-well plates. After filling each well with 100 µL of broth, dry test samples (*C. aromaticus* juice, ethanol, ethanol/water extracts) were dissolved in dimethyl sulfoxide (DMSO) at a final concentration of 20 mg/mL. These solutions were diluted and added to the first well of each microtiter line. Dilution in geometric progression was performed by transferring the mixture/dilution (100 µL) from the first to twelfth well. An aliquot (100 µL) was discarded from the twelfth well. The final concentration of the extracts used in the antimicrobial assay ranged from 4 to 0.002 mg/mL. The reference substances used were ampicillin (at 125 to 0.0625 µg/mL for bacteria and amphotericin B at 125 to 0.0625 µg/mL for yeast). The tests were incubated in adequate conditions, as described above. The end point was made by visual observation of growth. The MIC value was considered as the lowest sample’s concentration that prevented visible growth. In addition, 100 µL of suspension from each well without growth was inoculated in an agar plate to control the bacterial viability. After 48 h of incubation, the plates were tested for bacterial growth. The MBC (minimum bactericidal concentration) value was defined as the minimal concentration of the extracts necessary to kill the organisms [79].

### 4.5. Phytochemical Analysis of C. aromaticus Extracts

#### 4.5.1. Total Phenolic Content

The total phenolic content of the *C. aromaticus* extracts was determined according to the Folin–Ciocalteu spectrophotometric method. Gallic acid was used as a standard [80]. The reaction mixture (50 μL of Folin–Ciocalteu reagent, 50 μL of distilled water, and 50 μL of the extract at a concentration 1000 μg/mL) was mixed well in a test tube. Then, 50 μL Na_2_CO_3_ 10% solution was added, mixed again, and incubated for one hour at 40 °C in a water bath. The absorbance of the reaction mixture was measured at 765 nm. The TPC results are presented as mg of gallic acid equivalent (GAE) per gram of dry extract (mg GAE/g DE). The standard curve equation: y = 0.0136x + 0.0093 (R^2^ = 0.9879); each result was repeated three times.

#### 4.5.2. Total Flavonoid Content

The total flavonoid content (TFC) was estimated by the aluminum chloride (AlCl_3_) colorimetric method [80]. Quercetin was used as a standard. The reaction mixture consisted of 150 μL of AlCl_3_ (10% solution in methanol) and 150 μL of extract (500 μg/mL). The test tube was mixed well and incubated at room temperature for 10 min. The absorbance of the probe was measured at 300 nm. The results of the TFC were expressed as milligrams of quercetin equivalent (QE) per gram of dry extract (mg QE/g DE). All measurements were carried out in triplicate. The standard curve equation: y = 0.0039x + 0.0255 (R^2^ = 0.997).

#### 4.5.3. Sample Preparation for GC Analysis

First, 1 mL of hexane was added to weighed samples of *Coleus* juice (10 mg), ethanol/water extract (5 mg), and ethanol extract (10 mg). Then, the samples were sonicated for 5 min and filtered through 0.22 μm PTFE syringe filters.

#### 4.5.4. GC-FID Analysis

*C. aromaticus* extract samples were analyzed using an Agilent 8860 GC system equipped with an FID detector using HP-5 30 m × 0.25 mm i.d. × 0.25 µm film thickness capillary column (Agilent, Santa Clara, CA, USA). The temperature program started at 60 °C and then increased by 3 °C/min to 246 °C. The temperature of the injector was 250 °C. while the temperature of the FID was 300 °C. The flow rate of nitrogen was 1 mL/min. The injection volume was 0.2 µL with split 1:20. The content of individual components was expressed as a peak area percent integrated by OpenLab CDS version 2.4 (Agilent, Santa Clara, CA, USA).

#### 4.5.5. GC-MS Analysis

*C. aromaticus* extract samples of volatile components were identified using an Agilent 7000 Triple Quadrupole MSD coupled to a 5890A GC system (Agilent, Santa Clara, CA, USA). Analysis was performed on HP-5MS 30 m × 0.25 mm i.d. × 0.25 µm film thickness capillary column (Agilent, Santa Clara, CA, USA). The temperature program started at 60 °C and then increased by 3 °C/min to 246 °C. The temperature of the injector was 250 °C. The flow rate of helium was 1 mL/min. The injection volume was 0.2 µL with split 1:100. The transfer line temperature was 250 °C. The electron ionization potential was set at 70 eV, and the electron ionization (EI) source worked at 230 °C. The MS1 and MS2 quadrupoles’ temperature was 150 °C. Samples were analyzed in MS1 scan mode. The analytes were identified based on comparison with the data in Wiley Mass Spectral Data 11th edition. The data obtained were also compared with the literature data [81].

## 5. Conclusions

In our study, we estimated the biological activity of three different *C. aromaticus* extracts—ethanol, ethanol/water, and juice prepared from fresh leaves. The ethanol extract showed the strongest anticancer potency on human gastric cancer AGS and colorectal cancer HCT 116 cells, as well as the best antioxidant properties. The biological effect may result from the presence of phenolics and flavonoids, and the large amount of carvacrol in the ethanol extract composition. Thus, the plant has high therapeutic potential, both in cancer prevention and treatment. Further studies are planned to be carried out to fractionate the extract and determine which metabolites are responsible for these plant’s strong effects.

## Figures and Tables

**Table 1:** IC_50_ values of *C. aromaticus* extracts on AGS, HCT 116 cells, and fibroblasts HFF-1.

Cell Line	IC_50_ [µg/mL]			
	*C. aromaticus* Ethanol Extract	*C. aromaticus* Ethanol/Water Extract	*C. aromaticus* Juice	Oxaliplatin
AGS	4.94 ± 0.48	22.82 ± 1.82	n.r.	17.90 ± 1.20
HCT 116	24.99 ± 1.80	n.r.	n.r.	n.r.
Fibroblasts	45.31 ± 2.28	n.r.	n.r.	95.79 ± 3.24

n.r.—the IC_50_ values not reached in the used range of concentrations (10–150 µg/mL). The results were obtained via MTT assay in two independently performed experiments (in six repeats, *n* = 12). Oxaliplatin was used as a positive control.

**Table 2:** IC_50_ values of *C. aromaticus* extracts obtained in antioxidant tests: ABTS, DPPH, and molybdenum reducing power.

Assay	IC_50_ [µg/mL]			
	*C. aromaticus* Ethanol Extract	*C. aromaticus* Ethanol/Water Extract	*C. aromaticus* Juice	Ascorbic Acid
ABTS	13.34 ± 0.11	79.94 ± 0.55	148.90 ± 1.45	16.35 ± 0.97
DPPH	22.90 ± 1.30	418.40 ± 2.75	879.68 ± 2.55	20.15 ± 1.79
Molybdenum reducing power	290.17 ± 4.23	n.r.	n.r.	18.75 ± 1.54

Ascorbic acid was used as a standard; n.r.—not reached. The results were obtained in three independently performed experiments (in three repeats, *n* = 9).

**Table 3:** MIC and MBC values of *C. aromaticus* extracts on different microorganisms.

Microorganism	*C. aromaticus* Ethanol Extract		*C. aromaticus* Ethanol/Water Extract		*C. aromaticus* Juice		Ampicillin
	[mg/mL]						
	MIC	MBC	MIC	MBC	MIC	MBC	MIC
*Streptococcus* β-hemolytic group A PCM465	1	2	1	2	>2	>2	0.001
*Staphylococcus aureus* ATCC6538	2	2	>4	>4	>4	>4	0.00008
*Staphylococcus aureus* MRSA ATCC43300	1	>2	>2	>2	2	>2	0.01
*Enterococcus faecalis* ATCC51299	>4	>4	>4	>4	>4	>4	0.000125
*Corynebacterium diphtheriae* ZMF	4	4	>4	>4	>4	>4	0.001
*Escherichia coli* ATCC8739	>4	>4	>4	>4	>4	>4	0.0039
*Salmonella enterica* ATCC13076	>4	>4	>4	>4	>4	>4	0.0005
*Shigella sonnei* ATCC25931	2	>4	>4	>4	>4	>4	0.002
*Yersinia enterocolitica* ZMF4195	2	4	>4	>4	>4	>4	>0.5
*Helicobacter pylori* ATCC43504	2	4	>4	>4	>4	>4	0.0032
*Campylobacter jejuni* ZMF	4	>4	>4	>4	>4	>4	0.032
*Clostridium sporogenes* ATCC19404	1	>2	2	>2	2	>2	<0.0625
*Clostridium bifermentans* ATCC638	1	>2	>2	>4	1	2	0.016
*Clostridium perfringens* ATCC13124	<0.02	<0.02	<0.02	<0.02	>4	>4	<0.000016
*Listeria monocytogenes* PCM2191	>2	>2	>2	>2	>2	>2	0.016
*Lactobacillus paracasei* PCM2639	4	>4	>4	>4	>4	>4	>0.125
*Lactobacillus acidophilus* PCM2499	>4	>4	>4	>4	>4	>4	>0.125
*Candida albicans* ATCC26790	>4	>4	>4	>4	>4	>4	amphotericin B 0.001

**Table 4:** Phenolic and flavonoid content in *C. aromaticus* extracts expressed as mg of gallic acid equivalent (GAE) or quercetin equivalent (QE) in g of the dry extract (DE).

*C. aromaticus* Extract	Phenolic Content (mg GAE/g DE)	Flavonoid Content (mg QE/g DE)
Ethanol	75.87 ± 0.96	176.01 ± 3.58
Ethanol/water	35.13 ± 1.18	26.59 ± 0.52
Juice	27.19 ± 0.15	12.90 ± 1.54

Standard deviation is expressed as ±SD (*n* = 9).

**Table 5:** Content of detected volatile compounds in *C. aromaticus* extracts.

Peak	RT [min]	Area%	Compound	Molecular Formula
*C. aromaticus* ethanol extract				
1	7.441	2.77	1-ethylbutyl-hydroperoxide	C_6_H_14_O_2_
2	7.729	1.43	1-methylpentyl-hydroperoxide	C_6_H_14_O_2_
3	14.961	6.85	camphor	C_10_H_16_O
4	21.480	49.09	carvacrol	C_10_H_14_O
5	21.752	1.93	n/i	-
6	21.965	1.42	n/i	-
7	45.674	1.85	n/i	-
8	50.378	11.72	phytol	C_20_H_40_O
9	51.076	1.98	n/i	-
10	52.025	1.95	n/i	-
11	57.303	14.02	oleamide	C_18_H_35_NO
12	61.132	5	n/i	-
*C. aromaticus* ethanol/water extract				
1	7.470	6.49	1-ethylbutyl-hydroperoxide	C_6_H_14_O_2_
2	7.754	4.56	1-methylpentyl-hydroperoxide	C_6_H_14_O_2_
3	14.997	3.34	camphor	C_10_H_16_O
4	21.494	28.15	carvacrol	C_10_H_14_O
5	52.260	3.12	n/i	-
6	57.150	2.5	n/i	-
7	57.297	45.72	oleamide	C_18_H_35_NO
8	57.462	4.11	n/i	-
9	57.990	2.01	n/i	-
*C. aromaticus* juice				
1	7.433	4.34	1-ethylbutyl-hydroperoxide	C_6_H_14_O_2_
2	7.718	3.81	1-methylpentyl-hydroperoxide	C_6_H_14_O_2_
3	14.945	23.08	camphor	C_10_H_16_O
4	21.466	25.68	carvacrol	C_10_H_14_O
5	57.296	43.08	oleamide	C_18_H_35_NO

n/i—not identified, RT—retention time.

## Data Availability

The original contributions presented in this study are included in the article/Supplementary Materials. Further inquiries can be directed to the corresponding author.

## Supplementary Materials

The following supporting information can be downloaded at https://www.mdpi.com/article/10.3390/ph18111756/s1, Figure S1: The UV–Vis absorption spectra of *C. aromaticus* ethanol, ethanol/water extracts and juice (at a concentration of 0.1%) alone (**--------**) and after addition of copper (Cu^2+^) ions (**–––––**) at the concentration of 0.5 mM at pH 2 and 5.5. The shift of the maximum absorption after addition of Cu^2+^ to the extracts indicates forming of complexes (chelating ability of the extract). Red line (**–––––**) means spectrum of CuSO_4_ alone, blue arrow indicates the maximum absorption of the complex (the extract with Cu^2+^). EDTA was used as a control; Figure S2: Preliminary qualitative HPLC analysis of *C. aromaticus* ethanol, ethanol/water extracts and juice.
