# Supplementary material for: Coleus aromaticus Benth.—A Plant with Strong Anticancer and Antioxidant Potential In Vitro
Source: Pharmaceuticals (Basel). 2025 Nov 18;18(11):1756. doi: 10.3390/ph18111756 (PMC12655157; doi:10.3390/ph18111756)
Supplement: Supplementary file 1 [file pharmaceuticals-18-01756-s001.zip › pharmaceuticals-3922040-supplementary.pdf]

Supplementary Materials

Coleus aromaticus Benth.—A Plant with Strong Anticancer and Antioxidant Potential In Vitro

Justyna Stefanowicz-Hajduk [1,*], Anna Hering [1], Rafał Hałasa [2], Szymon Masiak [3], Karolina Turczyn [3], J. Renata Ochocka [1] and Monika Asztemborska [4]

[1] Department of Biology and Pharmaceutical Botany, Medical University of Gdańsk, 80-416 Gdańsk, Poland
[2] Department of Pharmaceutical Microbiology, Medical University of Gdańsk, 80-416 Gdańsk, Poland
[3] Faculty of Pharmacy, Medical University of Gdańsk, 80-210 Gdańsk, Poland
[4] Institute of Physical Chemistry, Polish Academy of Sciences, 01-224 Warsaw, Poland
* Correspondence: justyna.stefanowicz-hajduk@gumed.edu.pl

I. Cu^{2+} ion complexation by *C. aromaticus* extracts

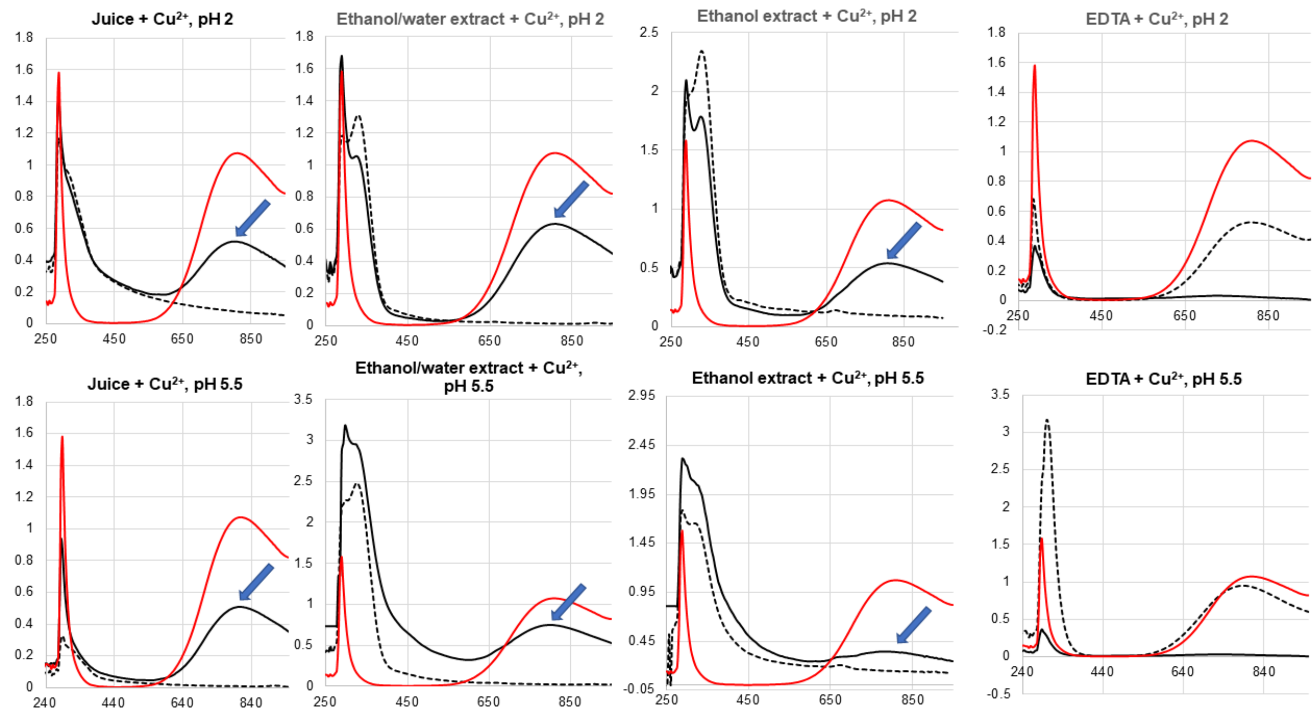

Figure S1. The UV–Vis absorption spectra of *C. aromaticus* ethanol, ethanol/water extracts and juice (at a concentration of 0.1%) alone (--------) and after addition of copper (Cu^{2+}) ions (———) at the concentration of 0.5 mM at pH 2 and 5.5. The shift of the maximum absorption after addition of Cu^{2+} to the extracts indicates forming of complexes (chelating ability of the extract). Red line (———) means spectrum of $CuSO_4$ alone, blue arrow indicates the maximum absorption of the complex (the extract with Cu^{2+}). EDTA was used as a control.

II. HPLC experiments

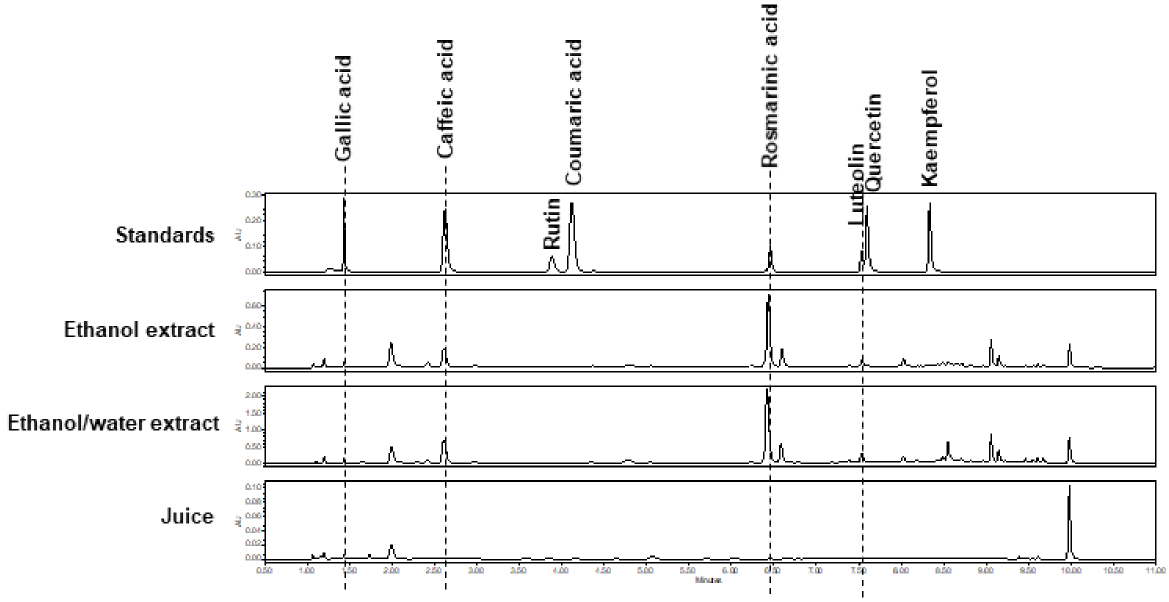

Figure S2. Preliminary qualitative HPLC analysis of *C. aromaticus* ethanol, ethanol/water extracts and juice.

Sample Preparation for HPLC Analysis

1 ml of methanol was added to weighed samples of *Coleus* juice (40 mg), ethanol/water extract (20 mg) and ethanol extract (20 mg). The samples were sonicated for 5 min and then filtered through 0.22 μm PTFE syringe filters.

HPLC Analysis

Chromatographic analyses were performed on Waters Acquity UPLC system equipped with PDA eλ detector. Acuity UPLC BEH C8 column (1.7 μm, 2.1 × 150 mm) (Waters, Ireland) was used. Column temperature was set to 40 ^{0}C. Analysis was carried with a programmed gradient of 0.1% formic acid in water (A) and 0.1% formic acid in acetonitrile (B). The flow rate was 0.3 mL/min. The gradient program was: 85% A and 15% B at 0 min to 10% A and 90% B at 10min, than 10% A and 90% B at 12 min, than back to the initial condition 85% A and 15% B at 12.5 min, than 85% A and 15 %B at 14 min. Chromatograms were recorded at 285, and 325 nm. The injection volume was 1 μL.
